# Evidence of HIV-1 adaptation to host HLA alleles following chimp-to-human transmission

**DOI:** 10.1186/1743-422X-6-164

**Published:** 2009-10-10

**Authors:** Nobubelo K Ngandu, Cathal Seoighe, Konrad Scheffler

**Affiliations:** 1 National Bioinformatics Node, Institute of Infectious Diseases and Molecular Medicine, Faculty of Health Sciences, University of Cape Town, Anzio Road, Observatory, 7925, South Africa; 2 School of Mathematics, Statistics and Applied Mathematics, National University of Ireland Galway, Ireland; 3 Computer Science Division, Dept of Mathematical Sciences, University of Stellenbosch, Private Bag X1, 7602 Matieland, South Africa

## Abstract

**Background:**

The cytotoxic T-lymphocyte immune response is important in controlling HIV-1 replication in infected humans. In this immune pathway, viral peptides within infected cells are presented to T-lymphocytes by the polymorphic human leukocyte antigens (HLA). HLA alleles exert selective pressure on the peptide regions and immune escape mutations that occur at some of the targeted sites can enable the virus to adapt to the infected host. The pattern of ongoing immune escape and reversion associated with several human HLA alleles has been studied extensively. Such mutations revert upon transmission to a host without the HLA allele because the escape mutation incurs a fitness cost. However, to-date there has been little attempt to study permanent loss of CTL epitopes due to escape mutations without an effect on fitness.

**Results:**

Here, we set out to determine the extent of adaptation of HIV-1 to three well-characterized HLA alleles during the initial exposure of the virus to the human cytotoxic immune responses following transmission from chimpanzee. We generated a chimpanzee consensus sequence to approximate the virus sequence that was initially transmitted to the human host and used a method based on peptide binding affinity to HLA crystal structures to predict peptides that were potentially targeted by the HLA alleles on this sequence. Next, we used codon-based phylogenetic models to quantify the average selective pressure that acted on these regions during the period immediately following the zoonosis event, corresponding to the branch of the phylogenetic tree leading to the common ancestor of all of the HIV-1 sequences. Evidence for adaptive evolution during this period was observed at regions recognised by HLA A*6801 and A*0201, both of which are common in African populations. No evidence of adaptive evolution was observed at sites targeted by HLA-B*2705, which is a rare allele in African populations.

**Conclusion:**

Our results suggest that the ancestral HIV-1 virus experienced a period of positive selective pressure due to immune responses associated with HLA alleles that were common in the infected human population. We propose that this resulted in permanent escape from immune responses targeting unconstrained regions of the virus.

## Background

Phylogenetic analysis indicates that the human immunodeficiency virus type 1 (HIV-1) originated from simian immunodeficiency virus infecting chimpanzees (SIVcpz) through a chimpanzee-to-human zoonotic transmission [1-4]. Until recently [5], the natural hosts of the virus, the chimpanzee, have been thought to remain asymptomatic throughout infection despite high viral loads [6-8] In humans, however, an increase in viral load is usually associated with progression to the acquired immuno-deficiency syndrome (AIDS) and subsequently death [9-13]. The causes of the difference in disease progression may involve either differences in the host and/or between the HIV-1 and the SIVcpz viruses.

A zoonotic (*i.e. *cross-species) event is expected to be accompanied by mutations that enable the pathogen to adapt to the new host environment, (e.g. as observed in a study by Baric et al [14]). Indeed, sequence changes have been identified in HIV-1 that are evidence of selective pressure associated with the genetics of the human host [15-17]. In particular, the human cytotoxic T-lymphocyte (CTL) immune response directed against foreign antigens plays a major role in exerting selective pressure on antigenic proteins, including those of HIV-1. The activation and characteristics of the immune responses against the virus have been found to differ remarkably between human and chimpanzee [7,18-20]: an elevated anti-HIV immune response upon infection is characteristic in humans, but the chimpanzee generally maintains a low level of immune activation. The human immune response may therefore exert higher selective pressure on the virus sequence compared to immune responses of the natural host. However, the virus is capable of overcoming the immune response, leading to AIDS.

The CTL immune response is mediated by Human Leukocyte Antigen (HLA) molecules that bind to endogenous antigenic peptides known as epitopes, and transport them to the surface of the infected cell for recognition by CTLs resulting in killing of the infected cell [21]. The HLA gene is highly polymorphic and each HLA molecule binds to peptides that contain specific sequence motif patterns (known as anchor residue motifs) [22,23]. For binding to occur between a peptide and the HLA binding groove, only limited amino acid variation at the main anchor positions of the peptide is allowed [21,24,25]. Successful binding, efficient transport and presentation of a peptide to a CTL depend on the presence of the appropriate anchor residue motif and the overall affinity between the HLA binding groove and the epitope [26,27]. The strength of selective pressure varies between specific CTL immune responses directed by different HLA alleles [28]. Some HLA molecules have been associated with immune escape mutations at anchor sites which enable the virus to adapt to the host, thus increasing viral load [8,29-31].

Investigation of the evolutionary dynamics of immune escape has focussed primarily on escape mutations that incur a fitness cost and consequently revert to wild type, upon transmission to a host that mounts different immune responses. This can result in a pattern of toggling between escape and wild-type amino acids that is detectable using evolutionary modelling [32]. In this study the focus is on escape mutations that do not incur a cost in terms of viral fitness. Such escape mutations do not experience selection pressure to revert to the wild-type state following transmission to a new host. Consequently, they are associated with episodic selection, rather than the ongoing rapid evolution associated with escape and reversion. Upon transmission to human, SIV is likely to have experienced selective pressure to escape from common human immune responses. Some of these escape mutations would not have had a significant effect on the fitness of the virus and thus would not have experienced strong selection to revert. Consequently, we hypothesized that the branch of the SIV-HIV-1 phylogenetic tree leading to the ancestor of the HIV-1 sequences would include evidence of episodic selection to escape from common HLA alleles.

To investigate the evidence of episodic selection for CTL escape along this branch, we predicted epitopes for HLA alleles, using the SIV consensus sequence to approximate the sequence that was transmitted to humans. We used a structure-based method that estimates the strength of binding between a viral amino acid sequence and an HLA molecule from amino acid pair-wise potentials for the epitope prediction. We selected regions where known anchor residue motifs were present and which had high binding affinity, limiting our analysis of selective pressure to these regions. Finally, we used models of codon sequence evolution to quantify the selective pressure, inferring positive selection from the ratio of nonsynonymous substitution rates (dN) to synonymous substitution rates (dS) for individual branches in a phylogeny. Branch-specific analysis of selective pressure enabled us to investigate selective pressure along the branch ancestral to the HIV sequences, and hence to study how HIV-1 adapted to the human host upon transmission from chimpanzee.

## Methods

### Sequence data

**Figure 1:**
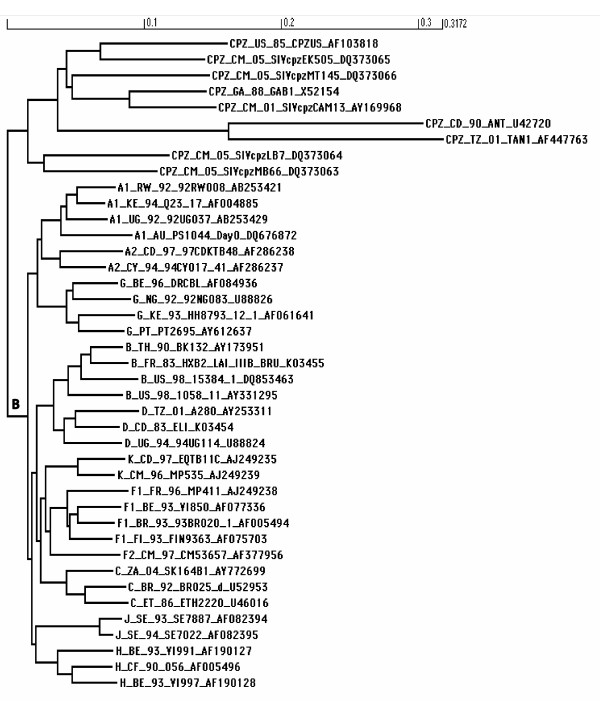
**The phylogenetic tree of the 32 HIV-1 group M reference genome sequences and 9 SIVcpz sequences from the Los Alamos sequence database **[33]. The chimpanzee sequence names start with 'CPZ' and the group M sequences start with the subtype name. The branch lengths are scaled in reference to the scale given at the top of the tree. The zoonosis event is located on the branch marked "B", referred to in the text as the HIV ancestral branch.

We downloaded an alignment of HIV-1 group M reference genome sequences and chimpanzee sequences from the Los Alamos database [33]. Previously, we found that some synonymous sites of the nucleotide sequence are highly conserved due to purifying selective pressure acting upon them, and that such conservation of synonymous sites can cause errors in the prediction of positive selection [34]. Therefore, in this study we removed the conserved regions identified in that study from the alignment (see Additional File 1). The resulting alignment consisted of 9 chimpanzee sequences and 32 HIV-1 sequences starting from codon 1 of the *gag *gene and ending with the *nef *stop codon, but excluding regions listed in Additional File 1. We used HyPhy [35] to build a phylogenetic tree (Figure 1) from the alignment using a neighbour joining method and the pairwise distances calculated using maximum likelihood.

### Predicting HLA binding regions

We used PREDEP [36], a structure-based method for predicting HLA binding peptides to determine potential binding regions across the genome. We used the consensus chimpanzee sequence to predict the best HLA binding regions because it has not been exposed to selective pressure resulting from human HLA and approximates the sequence that was transmitted to human. Consequently, it may be possible to detect epitopes in the chimpanzee sequence that were eliminated from HIV-1 shortly after transmission to humans. PREDEP does not require knowledge of known HLA-binding peptides. The program requires solved crystal structures of the HLA molecules as well as knowledge of amino acid residues on the HLA binding groove that interact with each position of the antigenic peptide sequence. Amino acid pair-wise potentials between the peptide and the amino acids in the HLA binding groove are calculated based on backbone and side-chain interactions.

A score for each HLA-peptide interaction is calculated as the sum of amino acid pair-wise potentials between each peptide residue and the interacting residues of the HLA binding groove. The lower the score, the better the peptide binds to the HLA binding groove, i.e., the higher the binding affinity. Peptides with strong binding affinities to the HLA molecule are most likely to be successfully presented to CTLs *in-vivo*. A test of PREDEP performance showed that 80% of the top 15 percentile best binders were known optimal HLA binding peptides [36]. In this study, we determined the binding energy of all possible peptides across the SIVcpz consensus sequence to each of the six HLA alleles with known crystal structures available in PREDEP. For each available HLA allele, we first selected peptide regions that had binding scores in the best 5% (a conservative threshold chosen to ensure minimal false positives) of those obtained across the chimpanzee sequence for that particular HLA allele. Next, we discarded regions that did not contain the amino acid residues known to give optimal binding at the major binding pockets, i.e. peptides that matched the anchor residue motifs of the HLA allele. For each HLA allele, we generated a new alignment consisting of only the sites in the potential binding regions identified for that allele for further analysis.

### Analysis of Selective pressure along the HIV-1 ancestral branch

We used the BranchAPriori [37] and GABranch [38] algorithms implemented in HyPhy [35] to analyse branch-specific selective pressure exerted by each HLA allele. We were specifically interested in selective pressure along the SIVcpz branch ancestral to the HIV-1 sequences (labelled 'B' in Figure 1, and referred to below as the HIV ancestral branch), because we expect that this reflects the evolution of the virus around the time of transmission from chimpanzees to human. We therefore investigated whether there is higher selective pressure on this branch at sites that are potential targets for each HLA allele under study. These two approaches calculate the average selective pressure acting upon all regions potentially targeted by an HLA allele, thus combining evidence from multiple sites. We expect that this should result in more powerful tests than can be obtained via site-specific analysis.

#### The BranchAPriori analysis

For each HLA-related alignment described in the previous section, we compared the selective pressure along the HIV ancestral branch to the rest of the branches in the tree using the BranchAPriori algotithm. The program outputs a p-value derived from the difference in the log likelihood between the null and the alternative models. The null model assumes a single global dN/dS ratio (ω) across the tree; in the alternative model, ω is allowed to have a different value for the HIV ancestral branch.

The *a priori *analysis has the disadvantage of assuming that all the branches in the rest of the tree are under uniform selective pressure. This could result in the analysis having reduced power, for instance when there is strong among-branch heterogeneity of selective pressure in the rest of the tree [39].

#### The GABranch analysis

In order to construct a more realistic null model, we therefore considered models that allow selective pressure to vary across all branches of the tree. We used GABranch [38], a genetic algorithm implemented in HyPhy, to infer branch-specific selective pressure across the entire phylogeny of SIV and HIV-1 sequences and determine, for each of the potential HLA binding regions, whether it evolved under positive selection in the HIV ancestral branch.

**Figure 2:**
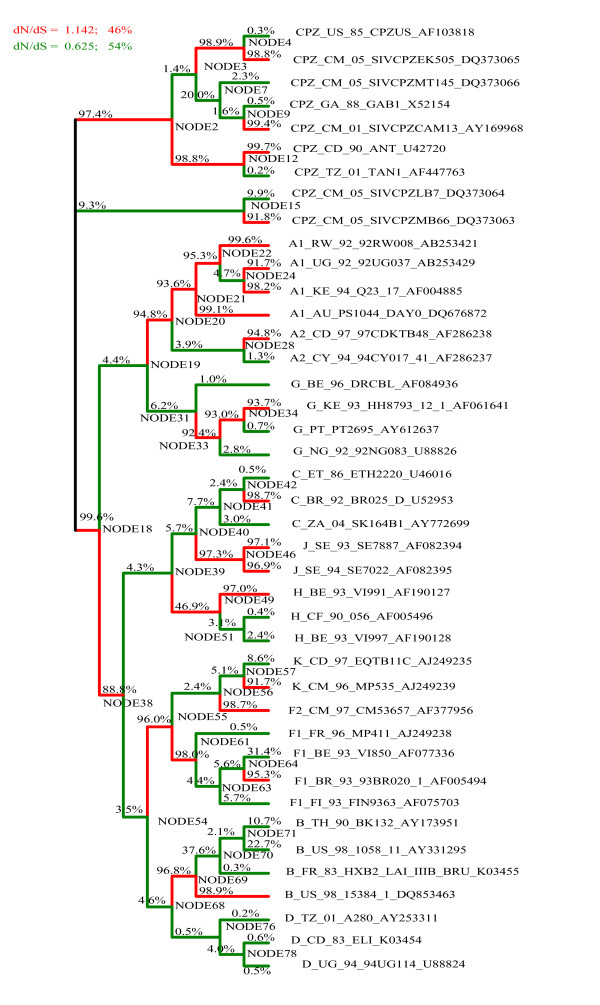
Branch-by-branch selective pressure for regions predicted to be targeted by HLA-A*0201. The ω classes for each branch are shown in colours given in the legend, along with the ω value for each class and the percentage of branches falling in each category. The percentage of models that support dN>dS are written above each branch.

**Figure 3:**
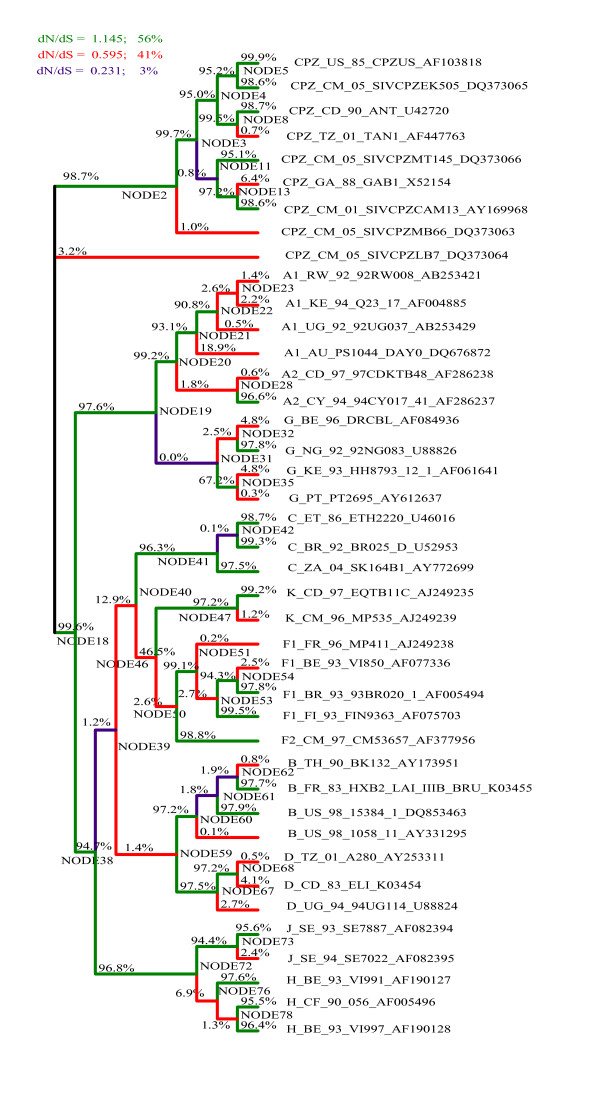
Branch-by-branch selective pressure for regions predicted to be targeted by HLA-A*6801. The ω classes for each branch are shown in colours given in the legend, along with the ω value for each class and the percentage of branches falling in each category. The percentage of models that support dN>dS are written above each branch.

**Figure 4:**
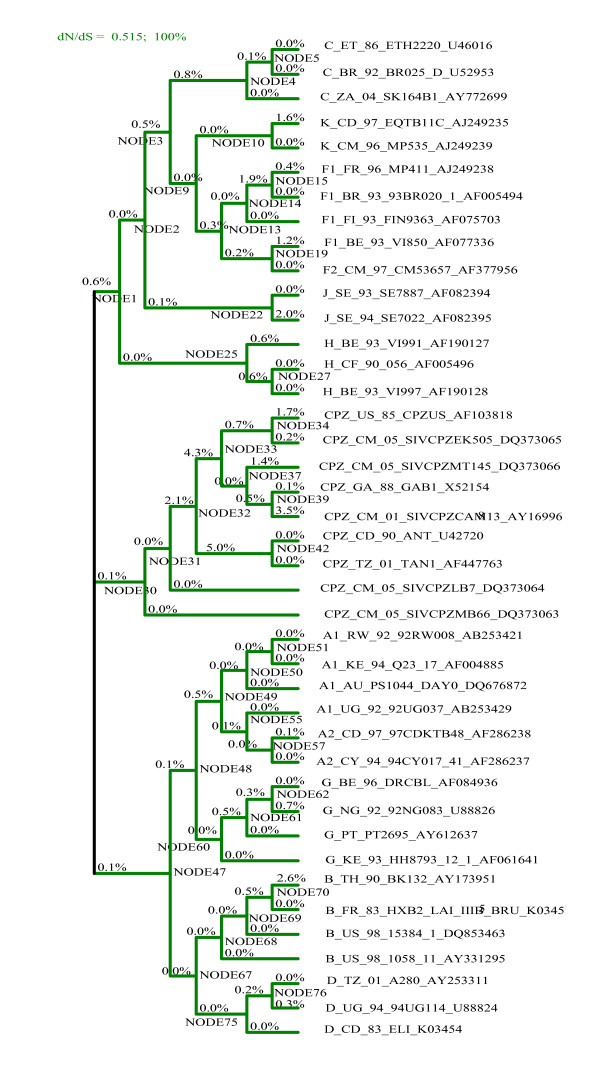
Branch-by-branch selective pressure for regions predicted to be targeted by HLA-B*2705. The ω classes for each branch are shown in colours given in the legend, along with the ω value for each class and the percentage of branches falling in each category. The percentage of models that support dN>dS are written above each branch.

As input, the GABranch analysis requires an underlying nucleotide model - we determined the best fitting nucleotide model using a maximum likelihood-based tool available in HyPhy [35]. GABranch then searches through a range of possible codon models with varying dN/dS rate classes, starting with a single rate model, i.e. a model that assumes uniform selection across all the branches of the tree. It tests models with more than one rate class, with the evolutionary rate of each branch being assigned to the best fitting rate class. An Akaike Information Criterion (AIC) value is calculated for each model, based on its fit to the data compared to the single rate model. The model with the best fit to the data, as indicated by the lowest AIC score, is selected and each branch is assigned to a dN/dS rate class (indicated on the phylogeny in figures 2, 3 and 4). Additionally, GABranch provides the proportion of tested models that show support for dN > dS for each branch.

## Results

### Prediction of HLA binding regions in the chimp sequence

Only six HLA alleles with solved crystal structures (given in Table 1) were available for analysis within the PREDEP program. Of these six, only HLAs A*0201, A*6801 and B*2705 showed strong binding to regions of the SIVcpz consensus sequence, that is, regions with scores that were within the top five percentile and also contained the preferred anchor residue motifs. The total length of the overlapping peptide regions predicted to be the best binders for each HLA molecule across the chimp genome are given in Table 2.

**Table 1:** HLA class 1 alleles with available crystal structure

**HLA allele**	**Allele frequency in African population**
A*0201	16%

A*6801	10%

B*2705	1%

B*3501	5%

B*5301	5%

B8	5%

HLA alleles with solved crystal structures that are available for analysis in the PREDEP program, with allele frequencies as estimated in [42].

**Table 2:** Sequence data for the regions predicted to have potential HLA binding peptides

**HLA allele**	**Anchor residue motif^1^**	**Predicted HLA binding regions**
A*0201	.[AILTVM]...... [AILTVM]	395 codons

A*6801	[AILTVM]...... [RK].	345 codons

B*2705	.[RK]...... [LFYRKHMI].	148 codons

^1 ^The anchor residue motifs were predicted from HLA-peptide structural conformations in PREDEP, residues in square brackets are the most preferred at the specific anchor site and the dots represent any other amino acid.

### BranchAPriori analysis of differential selection between the HIV and SIV lineages

We used the BranchAPriori analysis [39] to test, in the predicted binding region for each HLA allele, for evidence of differential selective pressure between the HIV ancestral lineage and the rest of the tree branches. For HLA A*0201 and HLA A*6801, the nonsynonymous-synonymous rate ratio ω was higher in the HIV ancestral branch than in the rest of the tree (Table 3). The difference was significant only for HLA A*6801, with a ω of 3.5 in the HIV ancestral branch and 1.6 in the rest of the tree (p value = 0.04). Selective pressure acting along the HIV ancestral branch for sites associated with A*0201 (ω = 2.5) was also high, but failed to differ significantly from the rest of the tree (ω = 1.2, p value = 0.08). For sites associated with B*2705 there was no significant difference between the HIV ancestral branch and the rest of the tree, with no indication of strong selective pressure in either case (ω = 1.0 in the HIV ancestral branch and 1.1 in the rest of the tree, p value = 0.65).

**Table 3:** BranchApriori ω values for the HIV ancestral branch and the rest of the tree

**Allele**	**HIV ancestral branch ω**	**Rest of the tree ω**	**p-value**
A*0201	2.5	1.2	0.08

A*6801	3.5	1.6	0.04

B*2705	1.0	1.1	0.65

### Branch-by-branch analysis of selective pressure using the GABranch algorithm

We ran the GABranch analysis on the sequence alignments of predicted binding regions from each HLA allele. The ω rate categories for the best fitting model as well as the number of branches assigned to each rate category are given in Table 4. Also shown, are the model-averaged values obtained for ω and estimated probabilities that ω>1 on the HIV ancestral branch. The proportion of models that have support for dN>dS for each branch is given in Figures 2, 3 and 4. The mean omega values and model support data for all 79 branches of the three trees are given in Additional files 2 (A*0201), 3 (A*6801) and 4 (B*2705). The tree in Figure 1 was made from the HIV and SIV full length sequences before selecting binding regions for each HLA allele, while those of Figures 2, 3 and 4 were generated from the screened alignments of binding regions for individual HLA alleles. We therefore do not expect these trees to have exactly the same topology.

**Table 4:** The best fitting models and model-averaged results obtained by the GABranch analysis

**HLA allele**	**Best fitting model: ω rate classes (number of branches)^1^**	**Model-averaged ω for HIV ancestral branch^2^**	**Model-averaged Prob(dN>dS)^3 ^for HIV ancestral branch**
A*0201	1.14 (33), 0.62 (46)	1.14	0.996

A*6801	1.15 (42), 0.60 (31), 0.23(6)	1.15	0.996

B*2705	0.52 (79)	0.52	0.06

^1^For each rate class, the value of ω is indicated followed by the number of branches (out of a total of 79) in brackets. ^2^The ω values are means over results from all the models. ^3^Prob (dN>dS) is the fraction of models that show support for dN>dS.

For HLA A*6801 (Figure 3), positive selective pressure was inferred along the HIV ancestral branch (ω = 1.15). A very high proportion of the tested models (0.996) supported dN>dS along this branch. We also found positive selective pressure along the HIV ancestral branch for HLA A*0201 (ω = 1.14, Figure 2) and again 0.996 of models supported for dN>dS. The best fitting model for the HLA B*2705 predicted binding sites had only a single rate class under weak purifying selection, and support for dN>dS was not obtained on any branch of the phylogeny (Figure 4). These results are consistent with those of the BranchAPriori analysis and suggest that HLA A*0201 and HLA A*6801 exerted positive selective pressure on the HIV-1 sequence in the period immediately following zoonosis, whereas HLA B*2705 did not exert strong selective pressure on the HIV-1 sequence at any point in the phylogeny.

## Discussion

The PREDEP program provides binding predictions for a limited number of HLA molecules with solved crystal structures and preferred binding anchor residue motifs that were predicted from HLA-peptide structural conformations. Only six such HLA alleles known to mediate cytotoxic T-lymphocyte immune responses are available for analysis. Amongst these, we only observed HLAs A*0201, A*6801 and B*2705 to bind strongly to some regions of the consensus SIVcpz genome. Our analysis was therefore restricted to selective pressure potentially exerted by each of these three alleles following the chimpanzee-to-human zoonosis event of HIV.

It is interesting that neither the *a priori *nor the GABranch analysis found evidence for positive selection in the HLA B*2705 alignment, whether along the ancestral HIV-1 branch or any other branch. This is surprising because B27 alleles have been associated with delayed progression to AIDS in HIV-1 infected individuals [40], which in turn is associated with persistent strong positive selection at specific sites [41]. Also, delayed progression is a result of reduced viral replication - this indicates that these sites are important for the fitness of the virus. One possibility that could explain the observed HLA B*2705 result is that it may have caused positive selection on only a few sites. Such selection is hard to detect because ω is averaged over all sites of the sequence. Selection may also have been weak due to the fact that this is a rare HLA allele (1%) [42].

In the HLA A*0201 dataset, both the *a priori *and the GABranch analyses inferred positive selection on the HIV-1 ancestral branch, with very high support for dN>dS. Of the alleles available for analysis in this study, HLA A*0201 is the most frequent in African populations (see Table 1). It is also the most frequent HLA allele in Caucasian populations and many studies have been carried out to determine its effect on HIV disease progression [42]. Even though the allele recognizes immunodominant peptide regions of the HIV-1 sequence, it fails to exert strong selective pressure on some virus peptides [43]. Some studies have also shown that the outcome of an immune response does not only depend on the HLA molecule but also on the specific peptide sequences that are targeted [44-48]. Our results suggest that immune escape mutations that occurred for HLA A*0201 mediated CTL responses may have been selected for in the period immediately following zoonosis. If these adaptations subsequently became fixed in the viral population they would no longer be under diversifying selection today.

HLA A*6801 (another common allele in African populations) appears to have exerted strong selective pressure on the HIV-1 ancestral branch compared to the rest of the tree. High support (99.6% of the tested models) for ω > 1 was observed at the ancestral HIV branch. This allele has anchor residue motif restrictions that are shared within the HLA A3 supertype, the second most frequent supertype in the human population [49]. The HLA A*6801 allele itself targets the Tat protein, which is expressed in the early stages of the HIV-1 lifecycle, and CTL responses to this protein cause a significant reduction in disease progression rate [50]. Escape mutations from the CTL immune response have also been identified within Tat at the population level, causing reduced viral load [51,52]. The virus may have adapted well to the A*6801 responses early after the cross-species transmission event at sites that do not affect the replication of the virus. The recently observed association with a reduction in viral load indicates that there were also functionally important sites contained in A*6801 epitopes - this would have made it difficult for these regions to adapt to the immune response.

## Conclusion

This is the first study that analyses HLA-associated selective pressure following the transmission from chimpanzee to human across all potential target sites of the HIV-1 genome. We identified regions of the HIV-1 sequence that were initially targeted by the CTL immune response immediately after the cross-species transmission of HIV-1 from chimpanzee to human using the chimpanzee consensus sequence. Of the six HLA alleles with crystal structures available for analysis, we found strong binding regions; this could imply successful immune responses *in vivo*, for HLAs A*0201, A*6801 and B*2705. We determined the average extent of selective pressure exerted by each HLA allele along the branch leading to HIV-1 sequences. This branch represents the sequences that first encountered human immune response-directed selective pressure immediately following the zoonosis event. Our results suggest that HIV-1 adapted to CTL responses directed by HLAs A*6801 and A*0201, which are amongst the most common HLA genotypes in African populations (Table 1). It is therefore likely that the virus was frequently exposed to selective pressure exerted by common immune responses during initial exposure to the human host following transmission of the virus from chimpanzees. As observed from the results, we did not find evidence for strong selective pressure exerted by the HLA B*2705, which has extremely low frequencies in the African populations (Table 1) [53,54].

In this study we focussed specifically on epitopes that we infer were likely to have been present in the viral sequence that first infected humans. We propose that the selection we observe at these positions along the branch of the phylogenetic tree leading to all of the HIV-1 sequences reflects episodic selection to evade human cytotoxic immune responses. Episodic selection has been proposed to be an important aspect of cross-species pathogen transmission and, in fact, observed in a laboratory setting previously [14]. However, this is the first time, to our knowledge, that evidence has been presented of transient positive selection associated with human immune responses against unconstrained regions of the virus shortly after transmission to human.

## Competing interests

The authors declare that they have no competing interests.

## Authors' contributions

NKN performed the analysis, interpreted the results and wrote the manuscript. CS conceived and supervised the study and edited the manuscript. KS supervised and co-wrote the manuscript. All authors read and approved the final manuscript.

## Supplementary Material

Additional file 1 **Regions of the HIV-1 sequence excluded from analysis**. Regions of the HIV-1 genome with highly conserved synonymous sites under purifying selective pressure reported in our previous study [34]. Co-ordinates are adapted from the HXB2 numbering system. No conserved synonymous sites were found along the *vif *gene region. Click here for file

Additional file 2 Model Averaged Branch dN/dS for HLA A*0201. The statistical distribution of dN/dS values for the HLA A*0201 binding regions along each branch of the tree, obtained via AIC-based model averaging. Branches with high model-averaged support for dN>dS are shown in bold. The HIV ancestral branch is Node 18. Click here for file

Additional file 3 Model Averaged Branch dN/dS for HLA A*6801. The statistical distribution of dN/dS values for the HLA A*6801 binding regions along each branch of the tree, obtained via AIC-based model averaging. Branches with high model-averaged support for dN>dS are shown in bold. The HIV ancestral branch is Node 18. Click here for file

Additional file 4 Model Averaged Branch dN/dS for HLA B*2705. The statistical distribution of dN/dS values for the HLA B*2705 binding regions along each branch of the tree, obtained via AIC-based model averaging. No model-averaged support for dN>dS was observed in any of the branches. Click here for file
