# Supplementary material for: Evidence of HIV-1 adaptation to host HLA alleles following chimp-to-human transmission
Source: Virol J. 2009 Oct 10;6:164. doi: 10.1186/1743-422X-6-164 (PMC2765438; doi:10.1186/1743-422X-6-164)
Supplement: Additional file 1 — Regions of the HIV-1 sequence excluded from analysis. Regions of the HIV-1 genome with highly conserved synonymous sites under purifying selective pressure reported in our previous study [34]. Co-ordinates are adapted from the HXB2 numbering system. No conserved synonymous sites were found along the vif gene region. [file 1743-422X-6-164-S1.DOC]

| **HIV-1 Gene region** | **Sites that are conserved at the nucleotide level** |
| --- | --- |
| *gag* | 793 – 807  898 – 903  985 – 996  1309 – 1314 |
| *pol* | 4092 – 4094  4764 – 4790  4864 – 4866  4926 – 4937 |
| *vif* | - |
| *vpr* | 5769 – 5777  5794 - 5805 |
| *tat* | 5855 – 5863  5957 - 5968 |
| *vpu* | 6101 – 6106  6143 – 6151  6167 - 6178 |
| *env* | 7656 – 7667  7834 – 7842  8349 – 8354  8376 – 8378 |
| *nef* | 9067 – 9086  9087 – 9093  9183 – 9192  9391 – 9399 |
