# Supplementary material for: Evidence of HIV-1 adaptation to host HLA alleles following chimp-to-human transmission
Source: Virol J. 2009 Oct 10;6:164. doi: 10.1186/1743-422X-6-164 (PMC2765438; doi:10.1186/1743-422X-6-164)
Supplement: Additional file 2 — Model Averaged Branch dN/dS for HLA A*0201. The statistical distribution of dN/dS values for the HLA A*0201 binding regions along each branch of the tree, obtained via AIC-based model averaging. Branches with high model-averaged support for dN>dS are shown in bold. The HIV ancestral branch is Node 18. [file 1743-422X-6-164-S2.DOC]

| **Branch Name** | **Mean** | **Std.Dev.** | **2.5%** | **Median** | **97.5%** | **Prob{dN>dS}†** |
| --- | --- | --- | --- | --- | --- | --- |
| CPZ_US_85_CPZUS_AF103818 | 0.630 | 0.027 | 0.612 | 0.628 | 0.648 | 0.003 |
| **CPZ_CM_05_SIVCPZEK505_DQ373065** | 1.113 | 0.055 | 1.046 | 1.120 | 1.162 | **0.988** |
| **Node4** | 1.113 | 0.054 | 1.047 | 1.120 | 1.163 | **0.989** |
| CPZ_CM_05_SIVCPZMT145_DQ373066 | 0.639 | 0.070 | 0.612 | 0.628 | 0.685 | 0.023 |
| CPZ_GA_88_GAB1_X52154 | 0.630 | 0.032 | 0.612 | 0.628 | 0.649 | 0.005 |
| **CPZ_CM_01_SIVCPZCAM13_AY169968** | 1.116 | 0.044 | 1.051 | 1.120 | 1.163 | **0.994** |
| Node9 | 0.637 | 0.059 | 0.612 | 0.628 | 0.681 | 0.016 |
| Node7 | 0.724 | 0.186 | 0.616 | 0.632 | 1.119 | 0.200 |
| Node3 | 0.634 | 0.055 | 0.611 | 0.628 | 0.652 | 0.014 |
| **CPZ_CD_90_ANT_U42720** | 1.117 | 0.034 | 1.053 | 1.120 | 1.163 | **0.997** |
| CPZ_TZ_01_TAN1_AF447763 | 0.629 | 0.023 | 0.611 | 0.628 | 0.649 | 0.002 |
| **Node12** | 1.113 | 0.052 | 1.046 | 1.120 | 1.161 | **0.988** |
| **Node2** | 1.107 | 0.081 | 0.984 | 1.119 | 1.163 | **0.974** |
| CPZ_CM_05_SIVCPZLB7_DQ373064 | 0.679 | 0.139 | 0.614 | 0.630 | 1.101 | 0.099 |
| CPZ_CM_05_SIVCPZMB66_DQ373063 | 1.078 | 0.133 | 0.632 | 1.116 | 1.159 | 0.918 |
| Node15 | 0.673 | 0.141 | 0.612 | 0.629 | 1.127 | 0.093 |
| **A1_RW_92_92RW008_AB253421** | 1.117 | 0.050 | 1.052 | 1.120 | 1.163 | **0.996** |
| A1_UG_92_92UG037_AB253429 | 1.078 | 0.135 | 0.628 | 1.116 | 1.161 | 0.917 |
| **A1_KE_94_Q23_17_AF004885** | 1.109 | 0.068 | 1.036 | 1.119 | 1.163 | **0.982** |
| Node24 | 0.651 | 0.104 | 0.612 | 0.628 | 1.114 | 0.047 |
| **Node22** | 1.099 | 0.144 | 0.629 | 1.119 | 1.163 | **0.953** |
| **A1_AU_PS1044_DAY0_DQ676872** | 1.115 | 0.055 | 1.048 | 1.120 | 1.163 | **0.991** |
| Node21 | 1.087 | 0.121 | 0.628 | 1.118 | 1.162 | 0.936 |
| A2_CD_97_97CDKTB48_AF286238 | 1.093 | 0.105 | 0.635 | 1.117 | 1.159 | 0.948 |
| A2_CY_94_94CY017_41_AF286237 | 0.634 | 0.054 | 0.611 | 0.628 | 0.652 | 0.013 |
| Node28 | 0.647 | 0.091 | 0.612 | 0.629 | 1.090 | 0.039 |
| Node20 | 1.093 | 0.110 | 0.631 | 1.118 | 1.162 | 0.948 |
| G_BE_96_DRCBL_AF084936 | 0.633 | 0.046 | 0.612 | 0.628 | 0.651 | 0.010 |
| G_KE_93_HH8793_12_1_AF061641 | 1.087 | 0.120 | 0.631 | 1.117 | 1.160 | 0.937 |
| G_PT_PT2695_AY612637 | 0.631 | 0.041 | 0.611 | 0.628 | 0.650 | 0.007 |
| Node34 | 9.241 | 285.455 | 0.628 | 1.117 | 1.162 | 0.930 |
| G_NG_92_92NG083_U88826 | 0.641 | 0.078 | 0.612 | 0.628 | 1.060 | 0.028 |
| Node33 | 1.084 | 0.133 | 0.624 | 1.119 | 1.163 | 0.924 |
| Node31 | 0.658 | 0.117 | 0.612 | 0.629 | 1.120 | 0.062 |
| Node19 | 0.649 | 0.097 | 0.612 | 0.629 | 1.091 | 0.044 |
| C_ET_86_ETH2220_U46016 | 0.630 | 0.033 | 0.611 | 0.628 | 0.649 | 0.005 |
| **C_BR_92_BR025_D_U52953** | 1.112 | 0.059 | 1.045 | 1.120 | 1.163 | **0.987** |
| Node42 | 0.639 | 0.075 | 0.611 | 0.628 | 0.675 | 0.024 |
| C_ZA_04_SK164B1_AY772699 | 0.642 | 0.079 | 0.612 | 0.629 | 1.059 | 0.030 |
| Node41 | 0.666 | 0.128 | 0.612 | 0.629 | 1.121 | 0.077 |
| **J_SE_93_SE7887_AF082394** | 17.020 | 398.613 | 0.645 | 1.119 | 1.163 | **0.971** |
| **J_SE_94_SE7022_AF082395** | 16.396 | 390.728 | 0.642 | 1.119 | 1.163 | **0.969** |
| **Node46** | 1.106 | 0.077 | 0.960 | 1.119 | 1.162 | **0.973** |
| Node40 | 0.656 | 0.112 | 0.612 | 0.629 | 1.121 | 0.057 |
| **H_BE_93_VI991_AF190127** | 1.105 | 0.077 | 0.895 | 1.118 | 1.161 | **0.970** |
| H_CF_90_056_AF005496 | 0.630 | 0.030 | 0.612 | 0.628 | 0.649 | 0.004 |
| H_BE_93_VI997_AF190128 | 0.640 | 0.073 | 0.612 | 0.628 | 0.677 | 0.024 |
| Node51 | 0.643 | 0.084 | 0.612 | 0.628 | 1.081 | 0.031 |
| Node49 | 0.855 | 0.236 | 0.618 | 0.645 | 1.142 | 0.469 |
| Node39 | 0.647 | 0.102 | 0.611 | 0.628 | 1.098 | 0.043 |
| K_CD_97_EQTB11C_AJ249235 | 0.669 | 0.130 | 0.612 | 0.629 | 1.100 | 0.086 |
| K_CM_96_MP535_AJ249239 | 1.079 | 0.134 | 0.630 | 1.118 | 1.162 | 0.917 |
| Node57 | 0.653 | 0.108 | 0.612 | 0.629 | 1.114 | 0.051 |
| **F2_CM_97_CM53657_AF377956** | 1.112 | 0.057 | 1.044 | 1.120 | 1.162 | **0.987** |
| Node56 | 0.640 | 0.074 | 0.612 | 0.628 | 0.682 | 0.024 |
| F1_FR_96_MP411_AJ249238 | 0.630 | 0.036 | 0.611 | 0.628 | 0.649 | 0.005 |
| F1_BE_93_VI850_AF077336 | 0.778 | 0.217 | 0.616 | 0.634 | 1.128 | 0.314 |
| **F1_BR_93_93BR020_1_AF005494** | 1.096 | 0.104 | 0.636 | 1.119 | 1.162 | **0.953** |
| Node64 | 0.655 | 0.112 | 0.612 | 0.628 | 1.121 | 0.056 |
| F1_FI_93_FIN9363_AF075703 | 0.656 | 0.109 | 0.612 | 0.629 | 1.101 | 0.057 |
| Node63 | 0.649 | 0.100 | 0.611 | 0.628 | 1.110 | 0.044 |
| **Node61** | 1.109 | 0.071 | 1.032 | 1.120 | 1.163 | **0.980** |
| **Node55** | 1.100 | 0.097 | 0.638 | 1.119 | 1.163 | **0.960** |
| B_TH_90_BK132_AY173951 | 0.678 | 0.143 | 0.612 | 0.630 | 1.107 | 0.107 |
| B_US_98_1058_11_AY331295 | 0.736 | 0.196 | 0.614 | 0.632 | 1.127 | 0.227 |
| Node71 | 0.638 | 0.069 | 0.611 | 0.628 | 0.661 | 0.021 |
| B_FR_83_HXB2_LAI_IIIB_BRU_K03455 | 0.629 | 0.028 | 0.611 | 0.628 | 0.648 | 0.003 |
| Node70 | 0.808 | 0.230 | 0.614 | 0.636 | 1.138 | 0.376 |
| **B_US_98_15384_1_DQ853463** | 1.113 | 0.050 | 1.047 | 1.120 | 1.163 | **0.989** |
| **Node69** | 1.103 | 0.088 | 0.639 | 1.119 | 1.163 | **0.968** |
| D_TZ_01_A280_AY253311 | 0.627 | 0.027 | 0.611 | 0.628 | 0.647 | 0.002 |
| D_CD_83_ELI_K03454 | 0.631 | 0.036 | 0.611 | 0.628 | 0.649 | 0.006 |
| D_UG_94_94UG114_U88824 | 0.631 | 0.035 | 0.611 | 0.628 | 0.649 | 0.005 |
| Node78 | 0.647 | 0.092 | 0.612 | 0.629 | 1.088 | 0.040 |
| Node76 | 0.630 | 0.032 | 0.612 | 0.628 | 0.649 | 0.005 |
| Node68 | 0.650 | 0.099 | 0.612 | 0.629 | 1.092 | 0.046 |
| Node54 | 0.645 | 0.089 | 0.612 | 0.628 | 1.092 | 0.035 |
| Node38 | 1.065 | 0.155 | 0.625 | 1.116 | 1.163 | 0.888 |
| **Node18** | 1.117 | 0.037 | 1.052 | 1.120 | 1.163 | **0.996** |
