# Supplementary material for: Evidence of HIV-1 adaptation to host HLA alleles following chimp-to-human transmission
Source: Virol J. 2009 Oct 10;6:164. doi: 10.1186/1743-422X-6-164 (PMC2765438; doi:10.1186/1743-422X-6-164)
Supplement: Additional file 3 — Model Averaged Branch dN/dS for HLA A*6801. The statistical distribution of dN/dS values for the HLA A*6801 binding regions along each branch of the tree, obtained via AIC-based model averaging. Branches with high model-averaged support for dN>dS are shown in bold. The HIV ancestral branch is Node 18. [file 1743-422X-6-164-S3.DOC]

| **Branch Name** | **Mean** | **Std.Dev.** | **2.5%** | **Median** | **97.5%** | **Prob{dN>dS}†** |
| --- | --- | --- | --- | --- | --- | --- |
| **CPZ_US_85_CPZUS_AF103818** | 1.129 | 0.032 | 1.066 | 1.134 | 1.171 | **0.999** |
| **CPZ_CM_05_SIVCPZEK505_DQ373065** | 1.122 | 0.063 | 1.052 | 1.133 | 1.168 | **0.986** |
| **Node5** | 1.103 | 0.115 | 0.607 | 1.132 | 1.168 | **0.952** |
| **CPZ_CD_90_ANT_U42720** | 1.122 | 0.061 | 1.053 | 1.133 | 1.166 | **0.987** |
| CPZ_TZ_01_TAN1_AF447763 | 0.598 | 0.043 | 0.543 | 0.598 | 0.625 | 0.007 |
| **Node8** | 1.127 | 0.042 | 1.062 | 1.134 | 1.170 | **0.995** |
| Node4 | 1.102 | 0.120 | 0.602 | 1.132 | 1.169 | 0.950 |
| **CPZ_CM_05_SIVCPZMT145_DQ373066** | 1.103 | 0.111 | 0.617 | 1.132 | 1.157 | **0.951** |
| CPZ_GA_88_GAB1_X52154 | 0.628 | 0.120 | 0.549 | 0.600 | 1.087 | 0.064 |
| **CPZ_CM_01_SIVCPZCAM13_AY169968** | 1.122 | 0.065 | 1.052 | 1.134 | 1.170 | **0.986** |
| **Node13** | 1.114 | 0.090 | 0.619 | 1.133 | 1.170 | **0.972** |
| Node11 | 0.328 | 0.152 | 0.218 | 0.242 | 0.602 | 0.008 |
| **Node3** | 1.128 | 0.042 | 1.065 | 1.134 | 1.171 | **0.997** |
| CPZ_CM_05_SIVCPZMB66_DQ373063 | 0.600 | 0.056 | 0.541 | 0.598 | 0.628 | 0.010 |
| **Node2** | 1.124 | 0.077 | 1.054 | 1.134 | 1.171 | **0.987** |
| CPZ_CM_05_SIVCPZLB7_DQ373064 | 0.611 | 0.087 | 0.545 | 0.599 | 1.049 | 0.032 |
| A1_RW_92_92RW008_AB253421 | 0.601 | 0.064 | 0.542 | 0.598 | 0.630 | 0.014 |
| A1_KE_94_Q23_17_AF004885 | 0.605 | 0.082 | 0.541 | 0.599 | 0.646 | 0.022 |
| Node23 | 0.593 | 0.110 | 0.262 | 0.598 | 1.045 | 0.026 |
| A1_UG_92_92UG037_AB253429 | 0.591 | 0.061 | 0.532 | 0.598 | 0.625 | 0.005 |
| Node22 | 3.610 | 159.511 | 0.241 | 1.131 | 1.169 | 0.908 |
| A1_AU_PS1044_DAY0_DQ676872 | 0.695 | 0.194 | 0.588 | 0.604 | 1.119 | 0.189 |
| Node21 | 1.092 | 0.141 | 0.597 | 1.132 | 1.168 | 0.931 |
| A2_CD_97_97CDKTB48_AF286238 | 0.596 | 0.048 | 0.540 | 0.598 | 0.625 | 0.006 |
| **A2_CY_94_94CY017_41_AF286237** | 1.111 | 0.100 | 0.612 | 1.133 | 1.170 | **0.966** |
| Node28 | 0.600 | 0.078 | 0.538 | 0.598 | 0.633 | 0.018 |
| **Node20** | 7.889 | 259.986 | 1.059 | 1.134 | 1.171 | **0.992** |
| G_BE_96_DRCBL_AF084936 | 0.620 | 0.107 | 0.547 | 0.600 | 1.080 | 0.048 |
| **G_NG_92_92NG083_U88826** | 1.117 | 0.089 | 1.033 | 1.133 | 1.170 | **0.978** |
| Node32 | 0.599 | 0.100 | 0.334 | 0.598 | 0.656 | 0.025 |
| G_KE_93_HH8793_12_1_AF061641 | 0.619 | 0.116 | 0.541 | 0.599 | 1.127 | 0.048 |
| G_PT_PT2695_AY612637 | 0.594 | 0.041 | 0.540 | 0.598 | 0.623 | 0.003 |
| Node35 | 0.951 | 0.266 | 0.539 | 1.125 | 1.169 | 0.672 |
| Node31 | 0.285 | 0.106 | 0.208 | 0.236 | 0.560 | 0.000 |
| **Node19** | 1.117 | 0.089 | 1.011 | 1.133 | 1.170 | **0.976** |
| **C_ET_86_ETH2220_U46016** | 1.123 | 0.068 | 1.053 | 1.134 | 1.171 | **0.987** |
| **C_BR_92_BR025_D_U52953** | 1.126 | 0.052 | 1.059 | 1.134 | 1.171 | **0.993** |
| Node42 | 0.291 | 0.113 | 0.211 | 0.236 | 0.567 | 0.001 |
| **C_ZA_04_SK164B1_AY772699** | 1.116 | 0.084 | 0.999 | 1.133 | 1.170 | **0.975** |
| **Node41** | 1.109 | 0.107 | 0.605 | 1.133 | 1.169 | **0.963** |
| **K_CD_97_EQTB11C_AJ249235** | 1.127 | 0.072 | 1.059 | 1.134 | 1.171 | **0.992** |
| K_CM_96_MP535_AJ249239 | 0.595 | 0.072 | 0.533 | 0.598 | 0.628 | 0.012 |
| **Node47** | 1.114 | 0.095 | 0.615 | 1.133 | 1.170 | **0.972** |
| F1_FR_96_MP411_AJ249238 | 0.594 | 0.034 | 0.541 | 0.598 | 0.622 | 0.002 |
| F1_BE_93_VI850_AF077336 | 0.604 | 0.090 | 0.538 | 0.599 | 0.873 | 0.025 |
| **F1_BR_93_93BR020_1_AF005494** | 1.117 | 0.083 | 1.028 | 1.133 | 1.169 | **0.978** |
| Node54 | 1.097 | 0.138 | 0.561 | 1.133 | 1.169 | 0.943 |
| **F1_FI_93_FIN9363_AF075703** | 1.146 | 0.474 | 1.063 | 1.134 | 1.171 | **0.995** |
| Node53 | 0.607 | 0.090 | 0.540 | 0.599 | 1.083 | 0.027 |
| **Node51** | 1.125 | 0.061 | 1.059 | 1.134 | 1.171 | **0.991** |
| **F2_CM_97_CM53657_AF377956** | 1.123 | 0.061 | 1.055 | 1.134 | 1.171 | **0.988** |
| Node50 | 0.604 | 0.093 | 0.537 | 0.598 | 1.063 | 0.026 |
| Node46 | 0.839 | 0.266 | 0.549 | 0.616 | 1.155 | 0.465 |
| Node40 | 0.655 | 0.190 | 0.501 | 0.600 | 1.142 | 0.129 |
| B_TH_90_BK132_AY173951 | 0.597 | 0.053 | 0.541 | 0.598 | 0.626 | 0.008 |
| **B_FR_83_HXB2_LAI_IIIB_BRU_K03455** | 1.117 | 0.083 | 1.021 | 1.134 | 1.171 | **0.977** |
| Node62 | 0.316 | 0.163 | 0.218 | 0.238 | 0.601 | 0.019 |
| **B_US_98_15384_1_DQ853463** | 1.118 | 0.079 | 1.034 | 1.134 | 1.170 | **0.979** |
| Node61 | 0.309 | 0.156 | 0.212 | 0.237 | 0.599 | 0.018 |
| B_US_98_1058_11_AY331295 | 0.588 | 0.049 | 0.507 | 0.598 | 0.622 | 0.001 |
| **Node60** | 1.114 | 0.094 | 0.625 | 1.133 | 1.169 | **0.972** |
| D_TZ_01_A280_AY253311 | 0.596 | 0.045 | 0.540 | 0.598 | 0.624 | 0.005 |
| D_CD_83_ELI_K03454 | 0.615 | 0.106 | 0.541 | 0.599 | 1.113 | 0.041 |
| **Node68** | 1.115 | 0.095 | 0.617 | 1.133 | 1.171 | **0.972** |
| D_UG_94_94UG114_U88824 | 0.608 | 0.086 | 0.541 | 0.599 | 1.056 | 0.027 |
| **Node67** | 1.116 | 0.088 | 0.976 | 1.133 | 1.170 | **0.975** |
| Node59 | 0.602 | 0.064 | 0.542 | 0.599 | 0.631 | 0.014 |
| Node39 | 0.315 | 0.151 | 0.213 | 0.238 | 0.598 | 0.012 |
| **J_SE_93_SE7887_AF082394** | 1.104 | 0.124 | 0.600 | 1.133 | 1.171 | **0.956** |
| J_SE_94_SE7022_AF082395 | 0.603 | 0.090 | 0.537 | 0.599 | 0.948 | 0.024 |
| Node73 | 1.099 | 0.121 | 0.605 | 1.132 | 1.167 | 0.944 |
| **H_BE_93_VI991_AF190127** | 1.117 | 0.084 | 1.016 | 1.133 | 1.170 | **0.976** |
| **H_CF_90_056_AF005496** | 1.104 | 0.115 | 0.605 | 1.132 | 1.168 | **0.955** |
| **H_BE_93_VI997_AF190128** | 1.110 | 0.101 | 0.609 | 1.133 | 1.169 | **0.964** |
| Node78 | 0.595 | 0.077 | 0.527 | 0.598 | 0.629 | 0.013 |
| Node76 | 0.630 | 0.131 | 0.546 | 0.600 | 1.127 | 0.069 |
| **Node72** | 1.111 | 0.106 | 0.607 | 1.133 | 1.170 | **0.968** |
| Node38 | 1.100 | 0.123 | 0.597 | 1.132 | 1.169 | 0.947 |
| **Node18** | 1.128 | 0.040 | 1.064 | 1.134 | 1.171 | **0.996** |
