# Supplementary material for: Evidence of HIV-1 adaptation to host HLA alleles following chimp-to-human transmission
Source: Virol J. 2009 Oct 10;6:164. doi: 10.1186/1743-422X-6-164 (PMC2765438; doi:10.1186/1743-422X-6-164)
Supplement: Additional file 4 — Model Averaged Branch dN/dS for HLA B*2705. The statistical distribution of dN/dS values for the HLA B*2705 binding regions along each branch of the tree, obtained via AIC-based model averaging. No model-averaged support for dN>dS was observed in any of the branches. [file 1743-422X-6-164-S4.DOC]

| **Branch Name** | **Mean** | **Std.Dev.** | **2.5%** | **Median** | **97.5%** | **Prob{dN>dS}†** |
| --- | --- | --- | --- | --- | --- | --- |
| C_ET_86_ETH2220_U46016 | 0.506 | 0.020 | 0.468 | 0.515 | 0.529 | 0.000 |
| C_BR_92_BR025_D_U52953 | 0.506 | 0.025 | 0.468 | 0.515 | 0.529 | 0.000 |
| Node5 | 0.507 | 0.046 | 0.466 | 0.515 | 0.532 | 0.001 |
| C_ZA_04_SK164B1_AY772699 | 0.508 | 0.026 | 0.473 | 0.515 | 0.534 | 0.000 |
| Node4 | 0.516 | 0.060 | 0.475 | 0.515 | 0.652 | 0.008 |
| K_CD_97_EQTB11C_AJ249235 | 17.789 | 414.835 | 0.476 | 0.515 | 0.971 | 0.016 |
| K_CM_96_MP535_AJ249239 | 0.506 | 0.020 | 0.468 | 0.515 | 0.529 | 0.000 |
| Node10 | 0.504 | 0.041 | 0.466 | 0.515 | 0.531 | 0.000 |
| F1_FR_96_MP411_AJ249238 | 0.508 | 0.044 | 0.468 | 0.515 | 0.531 | 0.004 |
| F1_BR_93_93BR020_1_AF005494 | 0.508 | 0.032 | 0.468 | 0.515 | 0.533 | 0.000 |
| Node15 | 74.891 | 859.119 | 0.473 | 0.515 | 0.688 | 0.019 |
| F1_FI_93_FIN9363_AF075703 | 0.509 | 0.035 | 0.474 | 0.515 | 0.544 | 0.000 |
| Node14 | 0.505 | 0.027 | 0.468 | 0.515 | 0.529 | 0.000 |
| F1_BE_93_VI850_AF077336 | 0.539 | 0.115 | 0.477 | 0.515 | 0.936 | 0.012 |
| F2_CM_97_CM53657_AF377956 | 0.509 | 0.030 | 0.473 | 0.515 | 0.545 | 0.000 |
| Node19 | 9.339 | 297.041 | 0.468 | 0.515 | 0.529 | 0.002 |
| Node13 | 0.513 | 0.062 | 0.468 | 0.515 | 0.546 | 0.003 |
| Node9 | 0.524 | 0.070 | 0.475 | 0.515 | 0.800 | 0.000 |
| Node3 | 2.385 | 136.929 | 0.468 | 0.515 | 0.531 | 0.005 |
| J_SE_93_SE7887_AF082394 | 0.507 | 0.049 | 0.468 | 0.515 | 0.545 | 0.000 |
| J_SE_94_SE7022_AF082395 | 62.748 | 786.222 | 0.474 | 0.515 | 0.829 | 0.020 |
| Node22 | 0.516 | 0.053 | 0.475 | 0.515 | 0.657 | 0.001 |
| Node2 | 0.505 | 0.023 | 0.468 | 0.515 | 0.529 | 0.000 |
| H_BE_93_VI991_AF190127 | 0.512 | 0.069 | 0.468 | 0.515 | 0.534 | 0.006 |
| H_CF_90_056_AF005496 | 0.503 | 0.032 | 0.466 | 0.515 | 0.529 | 0.000 |
| H_BE_93_VI997_AF190128 | 0.508 | 0.024 | 0.475 | 0.515 | 0.542 | 0.000 |
| Node27 | 0.515 | 0.061 | 0.472 | 0.515 | 0.617 | 0.006 |
| Node25 | 0.505 | 0.029 | 0.468 | 0.515 | 0.529 | 0.000 |
| Node1 | 0.514 | 0.065 | 0.475 | 0.515 | 0.607 | 0.006 |
| CPZ_US_85_CPZUS_AF103818 | 0.566 | 0.135 | 0.481 | 0.515 | 0.979 | 0.017 |
| CPZ_CM_05_SIVCPZEK505_DQ373065 | 0.552 | 0.113 | 0.481 | 0.515 | 0.901 | 0.002 |
| Node34 | 0.520 | 0.138 | 0.474 | 0.515 | 0.607 | 0.007 |
| CPZ_CM_05_SIVCPZMT145_DQ373066 | 0.532 | 0.105 | 0.475 | 0.515 | 0.875 | 0.014 |
| CPZ_GA_88_GAB1_X52154 | 0.548 | 0.110 | 0.481 | 0.515 | 0.909 | 0.001 |
| CPZ_CM_01_SIVCPZCAM13_AY169968 | 0.547 | 0.138 | 0.475 | 0.515 | 1.036 | 0.035 |
| Node39 | 0.521 | 0.071 | 0.475 | 0.515 | 0.792 | 0.005 |
| Node37 | 0.507 | 0.029 | 0.473 | 0.515 | 0.532 | 0.000 |
| Node33 | 77.942 | 876.219 | 0.475 | 0.515 | 1.232 | 0.043 |
| CPZ_CD_90_ANT_U42720 | 0.514 | 0.039 | 0.475 | 0.515 | 0.648 | 0.000 |
| CPZ_TZ_01_TAN1_AF447763 | 0.512 | 0.043 | 0.468 | 0.515 | 0.565 | 0.000 |
| Node42 | 0.604 | 0.177 | 0.488 | 0.516 | 1.086 | 0.050 |
| Node32 | 38.390 | 614.142 | 0.473 | 0.515 | 0.916 | 0.021 |
| CPZ_CM_05_SIVCPZLB7_DQ373064 | 0.508 | 0.025 | 0.473 | 0.515 | 0.538 | 0.000 |
| Node31 | 0.514 | 0.059 | 0.468 | 0.515 | 0.714 | 0.000 |
| CPZ_CM_05_SIVCPZMB66_DQ373063 | 0.506 | 0.023 | 0.468 | 0.515 | 0.531 | 0.000 |
| Node30 | 0.512 | 0.049 | 0.472 | 0.515 | 0.546 | 0.001 |
| A1_RW_92_92RW008_AB253421 | 0.509 | 0.030 | 0.475 | 0.515 | 0.546 | 0.000 |
| A1_KE_94_Q23_17_AF004885 | 0.504 | 0.049 | 0.421 | 0.515 | 0.536 | 0.000 |
| Node51 | 0.505 | 0.029 | 0.468 | 0.515 | 0.529 | 0.000 |
| A1_AU_PS1044_DAY0_DQ676872 | 0.512 | 0.038 | 0.475 | 0.515 | 0.607 | 0.000 |
| Node50 | 0.506 | 0.026 | 0.468 | 0.515 | 0.531 | 0.000 |
| A1_UG_92_92UG037_AB253429 | 0.506 | 0.022 | 0.468 | 0.515 | 0.529 | 0.000 |
| A2_CD_97_97CDKTB48_AF286238 | 0.531 | 0.096 | 0.473 | 0.515 | 0.904 | 0.001 |
| A2_CY_94_94CY017_41_AF286237 | 0.504 | 0.032 | 0.461 | 0.515 | 0.528 | 0.000 |
| Node57 | 0.506 | 0.025 | 0.468 | 0.515 | 0.529 | 0.000 |
| Node55 | 8.063 | 274.786 | 0.468 | 0.515 | 0.529 | 0.001 |
| Node49 | 0.524 | 0.282 | 0.468 | 0.515 | 0.536 | 0.005 |
| G_BE_96_DRCBL_AF084936 | 0.505 | 0.025 | 0.468 | 0.515 | 0.529 | 0.000 |
| G_NG_92_92NG083_U88826 | 0.513 | 0.068 | 0.468 | 0.515 | 0.555 | 0.007 |
| Node62 | 18.052 | 418.486 | 0.468 | 0.515 | 0.531 | 0.003 |
| G_PT_PT2695_AY612637 | 0.506 | 0.022 | 0.468 | 0.515 | 0.529 | 0.000 |
| Node61 | 13.033 | 353.648 | 0.468 | 0.515 | 0.532 | 0.005 |
| G_KE_93_HH8793_12_1_AF061641 | 0.506 | 0.027 | 0.468 | 0.515 | 0.529 | 0.000 |
| Node60 | 0.506 | 0.024 | 0.468 | 0.515 | 0.531 | 0.000 |
| Node48 | 0.509 | 0.036 | 0.473 | 0.515 | 0.543 | 0.001 |
| B_TH_90_BK132_AY173951 | 0.561 | 0.225 | 0.480 | 0.515 | 1.006 | 0.026 |
| B_FR_83_HXB2_LAI_IIIB_BRU_K03455 | 0.502 | 0.032 | 0.444 | 0.515 | 0.527 | 0.000 |
| Node70 | 4.174 | 191.289 | 0.475 | 0.515 | 0.607 | 0.005 |
| B_US_98_15384_1_DQ853463 | 0.507 | 0.031 | 0.468 | 0.515 | 0.531 | 0.000 |
| Node69 | 0.490 | 0.075 | 0.188 | 0.514 | 0.528 | 0.000 |
| B_US_98_1058_11_AY331295 | 0.506 | 0.028 | 0.468 | 0.515 | 0.529 | 0.000 |
| Node68 | 0.509 | 0.031 | 0.474 | 0.515 | 0.549 | 0.000 |
| D_TZ_01_A280_AY253311 | 0.497 | 0.053 | 0.280 | 0.515 | 0.528 | 0.000 |
| D_UG_94_94UG114_U88824 | 0.510 | 0.054 | 0.468 | 0.515 | 0.532 | 0.003 |
| Node76 | 0.508 | 0.044 | 0.468 | 0.515 | 0.531 | 0.002 |
| D_CD_83_ELI_K03454 | 0.502 | 0.032 | 0.441 | 0.515 | 0.527 | 0.000 |
| Node75 | 0.509 | 0.029 | 0.474 | 0.515 | 0.544 | 0.000 |
| Node67 | 0.505 | 0.029 | 0.468 | 0.515 | 0.529 | 0.000 |
| Node47 | 0.513 | 0.050 | 0.475 | 0.515 | 0.633 | 0.001 |
